# Women Patrols and Women Police

**Published:** 1916-07-01

**Authors:** D. O. G. Peto

**Affiliations:** Director, Bristol Training School for Women Patrols and Police. 5 Belgrave Road. Tyndall's Park, Bristol.


*To the Editor of* THE HOSPITAL.

### Women Patrols and Women Police.

*To the Editor of* THE HOSPITAL.

SIR,—I should be extremely grateful if you would allow me, through your paper, to make an appeal for recruits on behalf of the new and important movement now spreading through the country—that of women patrols and women police. Women of education, tact, common-sense, and perseverance, with previous experience in some branch of social service, such as nursing, club work, health visiting, and so forth, are urgently needed to fill posts now offering as patrol-leaders, policewomen, etc. The course of training before appointment depends upon individual needs and qualifications, and the pay, after appointment, ranges from 30s. to £2 2s. a week. At a time like the present, when too much cannot be done to protect and stimulate the moral growth of our national girlhood, it would be disastrous to neglect the opportunity offered to women police and patrols; and if any of your readers wish to offer themselves for training, or to make further inquiries, I shall be very glad indeed to hear from them.—I am, yours truly,

D. O. G. PETO, Director,
Bristol Training School for Women Patrols and Police.

5 Belgrave Road, Tyndall's Park, Bristol.

June 27, 1916.

